# Detection of Exosomal PD-L1 RNA in Saliva of Patients With Periodontitis

**DOI:** 10.3389/fgene.2019.00202

**Published:** 2019-03-14

**Authors:** Jialiang Yu, Yusheng Lin, Xiao Xiong, Kai Li, Zhimeng Yao, Hongmei Dong, Zuojie Jiang, Dan Yu, Sai-Ching Jim Yeung, Hao Zhang

**Affiliations:** ^1^Department of Stomatology, The First Affiliated Hospital of Shantou University Medical College, Shantou, China; ^2^Cancer Research Center, Shantou University Medical College, Shantou, China; ^3^Institute of Precision Cancer and Pathology, Jinan University Medical College, Guangzhou, China; ^4^Department of Emergency Medicine, University of Texas MD Anderson Cancer Center, Houston, TX, United States; ^5^Department of Endocrine Neoplasia and Hormonal Disorders, University of Texas MD Anderson Cancer Center, Houston, TX, United States; ^6^Research Center of Translational Medicine, The Second Affiliated Hospital of Shantou University Medical College, Shantou, China

**Keywords:** immune checkpoint, exosomes, saliva, chronic periodontal disease, biomarker, disease stage

## Abstract

Periodontitis is the most prevalent inflammatory disease of the periodontium, and is related to oral and systemic health. Exosomes are emerging as non-invasive biomarker for liquid biopsy. We here evaluated the levels of programmed death-ligand 1 (*PD-L1*) mRNA in salivary exosomes from patients with periodontitis and non-periodontitis controls. The purposes of this study were to establish a procedure for isolation and detection of mRNA in exosomes from saliva of periodontitis patients, to characterize the level of salivary exosomal *PD-L1*, and to illustrate its clinical relevance. Bioinformatics analysis suggested that periodontitis was associated with an inflammation gene expression signature, that *PD-L1* expression positively correlated with inflammation in periodontitis based on gene set enrichment analysis (GSEA) and that *PD-L1* expression was remarkably elevated in periodontitis patients versus control subjects. Exosomal RNAs were successfully isolated from saliva of 61 patients and 30 controls and were subjected to qRT-PCR. Levels of *PD-L1* mRNA in salivary exosomes were higher in periodontitis patients than controls (*P* < 0.01). Salivary exosomal *PD-L1* mRNA showed significant difference between the stages of periodontitis. In summary, the protocols for isolating and detecting exosomal RNA from saliva of periodontitis patients were, for the first time, characterized. The current study suggests that assay of exosomes-based *PD-L1* mRNA in saliva has potential to distinguish periodontitis from the healthy, and the levels correlate with the severity/stage of periodontitis.

## Introduction

Periodontitis is one of the most prevalent disease in dentistry, impairing the integrity of the periodontium and leading ultimately to tooth loss. Periodontitis is a chronic inflammatory disease caused by microorganisms colonizing the dentogingival interface (Slots, 2013). Currently, the diagnosis of periodontitis was mostly based on clinical and radiographic evaluations without specific evaluation of the underlying inflammatory response (Buduneli and Kinane, 2011; Baeza et al., 2016). Measuring electrolyte concentration in gingival crevicular fluid (GCF), especially the concentration of sodium, potassium and calcium ions, reflects the clinical status of the periodontal tissues such that the pattern of concentrations of these ions may be used as a potential diagnostic marker for active periodontitis (Koregol et al., 2011).

Given the close relevance of inflammation to periodontitis, immune-regulatory factors have been explored as biomarker of periodontitis (Han et al., 2012; Kimak et al., 2015). Programmed death-ligand 1 (PD-L1), also known as the B7-H1 receptor, plays an important role in cell-mediated immune responses (Gianchecchi et al., 2013). PD-L1 regulates T cell activation and tolerance, and is able to inhibit activated T cell functions and survival (Kim et al., 2005). High expression of PD-L1 in host cells may contribute to the chronicity of inflammatory disorders (Zamani et al., 2016).

PD-L1 is involved in periodontitis. *Porphyromonas gingivalis* (*P. gingivalis*) is a keystone pathogen in chronic periodontitis (Groeger et al., 2017), and it induces expression of PD-L1 in malignant and non-malignant oral epithelial cells (Groeger et al., 2017). In periodontitis, P. gingivalis inhibits the synthesis of cytokines and increases humoral responses. This reduces the inflammatory responses related to T- and B-cell activation, and subsequent IFN-γ secretion by a subset of T cells. The T cells that secrete IFN-γ further suppress upregulation of programmed cell death-1 (PD-1)-receptor and its ligand PD-L1 on CD11b+-subset of T cells (Gaddis et al., 2013; Groeger et al., 2017). Interestingly, studies recently have demonstrated elevated PD-L1 levels in saliva from patients with oral cancers or salivary gland carcinoma (Aziz et al., 2015; Goncalves et al., 2015, 2017). Saliva-derived samples have been studied as a source of biomarkers for periodontitis (Baltacioglu et al., 2014; Recker et al., 2015).

Exosomes are small (30—100 nm) membrane-encapsulated vesicles containing nuclei acids and protein cargo, and secreted by eukaryotic cells into the circulation (Tkach and Thery, 2016). The contents of disease cell-derived exosomes may potentially serve as a source of disease biomarkers. Salivary exosomal contents have recently been investigated for diagnosis and prognosis of a wide range of diseases (Michael et al., 2010; Lau et al., 2013; Byun et al., 2015; Machida et al., 2015; Sivadasan et al., 2015; Kim et al., 2017; Zheng et al., 2017; Han et al., 2018). Although detection of *PD-L1* mRNA has been reported in periodontitis (Yuan et al., 2015; Zhang et al., 2016), it is unknown whether *PD-L1* mRNA can be detected in exosomes of saliva of periodontitis patients and whether the level of salivary exosomal *PD-L1* mRNA reflects disease status. We therefore focused on exosomes and saliva, and set to establish a protocol for isolation and detection of exosomes, and exosomal RNA in the saliva of periodontitis patients, and to characterize salivary exosomal PD-L1 as a potential marker for periodontitis.

## Materials and Methods

### Study Population

This study is a prospective observational investigation at the First Affiliated Hospital of Shantou University. From June, 2017 to June, 2018, 61 periodontitis patients and 30 control subjects were enrolled in the study with informed consent. Each recruited subject was inquired in detail about the medical history and accepted a thorough periodontal examination. Diagnosis of periodontitis was based on assessment of probing pocket depth (PD) and clinical attachment loss (CAL). Patients who fulfilled the requirement of generalized chronic periodontitis according to the classification by the American Academy of Periodontology in 2007 (Slots, 2013) were recruited to the periodontitis group after periodontal examination. The non-periodontitis control group was composed of subjects with no evidence of periodontitis after examination according to the above classification. Smoking and alcohol using status was recorded for all individuals. Exclusion criteria were age <18 years, inability to give informed consent, congenital malformation, chronic diseases (e.g., lip cancer, gingival cancer, carcinoma of the tongue, soft palate carcinoma, jaw bone cancers, cancers of the mouth floor carcinoma, oropharyngeal cancer, salivary gland carcinoma, maxillary sinus carcinoma, cancer occurs in facial ministry skin mucous membrane, epilepsy, cardiac disease, lung disease, renal disease, positive test for human immunodeficiency virus etc.), and history of systemic antibiotic treatment or dental prophylaxis in the previous 6 months. Written informed consents were obtained from all participants in accordance with the principles established by the Helsinki Declaration. This study was approved by the Institutional Review Board of Shantou University Medical College (SUMC) (Shantou, China) under IRB protocol number: #04-070.

### Clinical Evaluation

For each individual, PD, CAL and bleeding on probing (BP) values were measured with a periodontal probe. A single calibrated examiner assessed these clinical parameters. Control individuals (control group) did not present any sign or symptom compatible with periodontal disease (PD < 3 mm, CAL < 3 mm and no radiographic evidence of alveolar bone breakdown). On the other hand, the periodontitis group consisted of patients with mild, moderate or severe periodontitis (based on the Classification of Periodontal Diseases and Conditions of Armitage, 2007) (Slots, 2013) with at least one single-rooted tooth with a CAL ≥ 6 mm and probing depth ≥ 5 mm.

### Gingival Biopsies

Human gingivae were obtained from all periodontitis patients. Gingiva was harvested during tooth extractions for periodontal reasons in the Department of Stomatology of First Affiliated Hospital of Shantou University. Written informed consent and approval of the Ethics Committee of the SUMC were obtained.

### Human Saliva Collection

Saliva was collected in the morning (8 am to 10 am) from all subjects. During the collection period, subjects were instructed to refrain from eating, drinking or using oral hygiene products for at least 1 h prior to collection, and received no stimulation of salivation. After rinsing the mouth with water, each subject spat 3–5 mL saliva into a 35-mm dish. Subjects were reminded not to cough up mucus, and saliva was collected within 30 min from spitting. These saliva samples were pipetted into 1.5-mL tubes and were kept on ice during processing which did not exceed 60 min. The samples were then centrifuged at 3,000 ×*g* for 15 min at 4°C to remove cells and cellular fragments. As much supernatant as possible was collected into new 1.5-mL tubes and stored at -80°C.

### Gene Set Enrichment Analysis (GSEA)

Gene set enrichment analysis (GSEA; v2.09^1^) was performed to examine the association between gene sets and gene expression (Dong et al., 2017). Periodontitis gene expression profiles from an independent datasets (GSE16134) were collected from NCBI Gene Expression Omnibus (GEO) at http://www.ncbi.nlm.nih.gov/geo/. The expression levels of affected gingival tissue (*n* = 241) vs. unaffected gingival tissue (*n* = 69) from GSE16134 was ordered from high to low. We performed GSEA analysis to examine the correlation between periodontitis and the PD-L1 signature pathway, followed by the protocol available at the GSEA website^1^.

### Isolation of Salivary Exosomes

ExoQuick^TM^ exosomes precipitation solution was used for exosomes isolation according to the manufacturer’s instructions (System Biosciences, Mountain View, CA, United States). Briefly, ExoQuick-TC^TM^ solution was added to saliva at a 63/250 ratio and mixed by inverting the tubes several times. Exosomes were precipitated by refrigeration at 4°C overnight, and then collected by centrifugation twice at 1,500 ×*g* for 30 and 5 min, respectively, in order to remove the supernatant. Supernatant was discarded, and the pellet resuspended in 300 μL TRIZOL for RNA isolation or 20 μL PBS for protein isolation.

### RT-qPCR

Total RNA was extracted from exosomes and cell lysates using TRIzol reagent as per the manufacturer’s protocol (ZYMO RESEARCH). RNA was eluted with 25 μL of RNAse-free water. RT-qPCR was performed using an Absolute Blue QPCR SYBR Green Low ROX mix (Thermo Scientific) on an Applied Biosystems’ 7500 real-time PCR system. The Rn value (normalized reporter value) was the fluorescent signal from SYBR Green normalized to the signal of the passive reference dye for a given reaction. No-template and no-RT reactions were performed as negative controls. All assays were performed in 3 separate RTs followed by triplicate qPCR, and the results are shown as the average fold change relative to GAPDH which served as an internal control. Primers for RT-qPCR were:

GAPDH F: 5′-TGCACCACCAACTGCTTAGC-3′
GAPDH R: 5′-GGCATGGACTGTGGTCATGAG-3′
PD-L1 F: 5′-TGCCGACTACAAGCGAATTACTG -3′
PD-L1 R: 5′-CTGCTTGTCCAGATGACTTCGG-3′.

### Protein Isolation and Immunoblotting

Exosomal pellets were resuspended in PBS and re-pelleted by centrifugation and then extracted with RIPA buffer (Santa Cruz Biotechnology). Total protein lysates were prepared and analyzed by immunoblotting using anti-ALIX (Cat. No. 2171; Cell Signaling Technology, Beverly, MA, United States), anti-TSG101 (Cat. ab133586; Abcam, Cambridge, United Kingdom), anti-CD63 (Cat. sc-15363; Santa Cruz Biotechnology, CA, United States), anti-CD9 (Cat. sc-9148; Santa Cruz Biotechnology, CA, United States), anti-CD81 (Cat. ab109201; Abcam, Cambridge, United Kingdom), anti-Calnexin (Cat. No. 2433; Cell Signaling Technology, Beverly, MA, United States), anti-LC3 (Cat. No. 2775; Cell Signaling Technology, Beverly, MA, United States), anti-NLRP3 (Cat.19771-1-AP; Proteintech, United States) and anti-NLRP4 (Cat. NB100-56156; Novus Biologicals, United States) as described previously (Gan et al., 2016; Dong et al., 2017).

### Transmission Electron Microscopy

Following exosomes isolation, the pellet was washed in PBS, and then subjected to ultracentrifugation at 120,000 ×*g* for 70 min to re-pellet the exosomes. The exosomes pellets were resuspended in 30 μL PBS, and a 10 μL aliquot of the suspension loaded onto formvar carbon-coated grids and allow to stand for 5 min at room temperature. Next, the exosomes were fixed in 2% paraformaldehyde for 5 min at room temperature and washed thrice with PBS. Excess liquid was drained by gently touching the edge of the grid with clean filter paper. The grid was slightly touched onto a drop of 2% uranyl acetate for 1 min and embedded in a mixture of uranyl acetate (0.8%) and methyl cellulose (0.13%). Excess liquid was drained off, and then the grid was allowed to air dry for several minutes prior to examination under a transmission electron microscope (JEM-1400, Hitachi, Shiga, Japan) (Raposo and Stoorvogel, 2013).

### Nanoparticle Tracking Analysis

Total particles in human salivary samples were analyzed by nanoparticle tracking by the A & P Instrument Co., Ltd. (Guangzhou, China), using a NanoSight LM10 system (NanoSight Ltd., Amesbury, United Kingdom). Each sample was diluted in nanoparticle-free PBS and analyzed three times. Data was collected and analyzed using the nanoparticle tracking analysis (NTA) software (RRID: SCR_014239, version 2.3). All measurements were recorded at room temperature.

### Cell Culture

All cells were cultured in a sterile incubator maintained at 37°C with 5% CO2. ESCC cells and THP-1 cells were cultured in Dulbecco’s modified Eagle’s medium (Gibco, Paisley, United Kingdom) or RPMI-1640 medium (Gibco, Paisley, United Kingdom) supplemented with 10% heat-inactivated fetal bovine serum (Gibco, Paisley, United Kingdom), 10 mmol/L glutamine, 100 units/ml penicillin (Sigma, St. Louis, MO, United States), and 100 μg/ml streptomycin (Sigma). TE1 cells were provided by Dr. X.C. Xu (The University of Texas MD Anderson Cancer Center, Houston, TX, United States). For LC3 detection, TE1 cells were collected after starvation (i.e., without fetal bovine serum) for 6 h.

### Statistical Analysis

All statistical analyses were performed using the SPSS 19.0 statistical software package (SPSS Inc., Chicago, IL, United States) and Prism V6.01 (GraphPad). Summary statistics reporting means and standard errors were stated as appropriate. Statistical methods used included *t*-test, Pearson correlation and chi-square test.

## Results

### Bioinformatic Analysis of the Correlation Between Periodontitis and PD-L1

**FIGURE 1:**
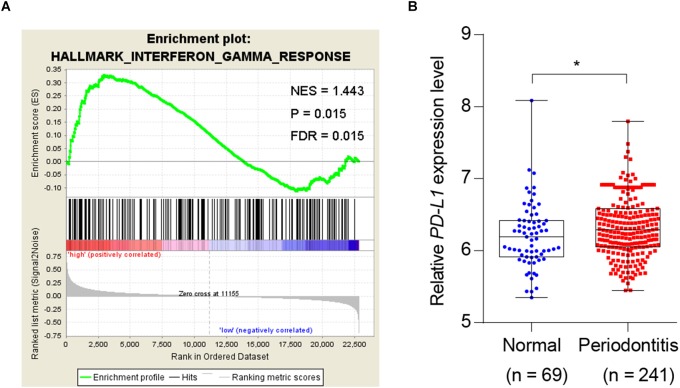
Periodontitis correlated with inflammation and *PD-L1* signatures. **(A)** PD-L1 gene signatures were analyzed by GSEA using the gene sets (GSE 16134) derived from periodontitis patients and normal subjects. FDR = 0.015. **(B)** Quantified results show the expression level of *PD-L1* in the gene sets (GSE 16134). Periodontitis patients (*n* = 241) compared with normal subjects (*n* = 69). Error bars indicate SEM. ^∗^*P*< 0.05 by Student’s *t-*test.

Given the association of periodontitis with inflammation, and the importance of PD-L1 in inflammatory immunity, we analyzed database to test whether periodontitis correlates with PD-L1. GSEA analysis revealed that periodontitis is positively associated with an inflammation signature, and PD-L1 is positively correlated with an inflammation signature in periodontitis (Figure 1A). Analysis of the GEO database (GSE16134) indicated a statistically significant elevation of PD-L1 level in periodontitis patients versus control subjects (Figure 1B). These data support the hypothesis that PD-L1 is positively relevant to periodontitis.

### Patient Demographics and Clinical Parameters

**FIGURE 2:**
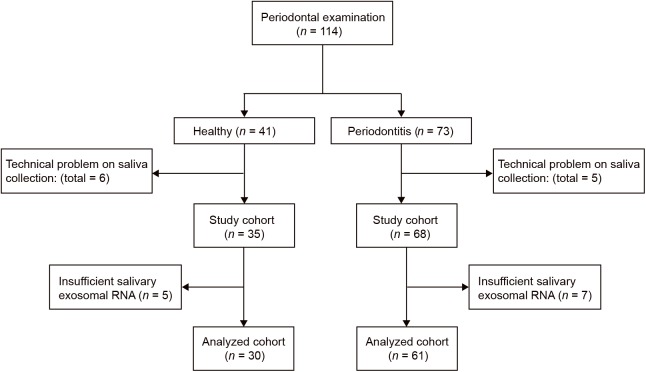
Flow diagram of periodontitis patients and control subjects. Participant numbers are accounted for by the flow diagram for the study.

This pilot study was conducted at Shantou University Medical College. Sixty-one periodontitis patients and 30 control subjects were enrolled in the study (Figure 2). With an average age of 51, 29 male and 32 female patients from the First Affiliated Hospital of Shantou University were recruited. There are also 30 control subjects (14 male and 16 female) were enrolled in this study with an average age of 52. Among demographic and clinicopathological characteristics, age, gender, tobacco use, alcohol use, hypertension and diabetes did not show any significant differences between the two groups (Table 1).

**Table 1:** The demographics and clinicopathological characteristics of the periodontitis patients and control subjects.

Variables	Control (*n* = 30) *n* (%)	Patient (*n* = 61) *n* (%)	*P-*value^∗^
**Age (years)**			
≤50	18 (31.6)	39 (68.4)	0.715
>50	12 (35.3)	22 (64.7)	
**Gender**			
Female	21 (39.6)	32 (60.4)	0.111
Male	9 (23.7)	29 (76.3)	
**Tobacco use**			
No	19 (32.2)	40 (67.8)	0.833
Yes	11 (34.4)	21 (65.6)	
**Alcohol use**			
No	20 (30.8)	45 (69.2)	0.481
Yes	10 (48.5)	16 (61.5)	
**Hypertension**			
No	23 (35.9)	41 (64.1)	0.353
Yes	7 (25.9)	20 (74.1)	
**Diabetes**			
No	27 (36.5)	47 (63.5)	0.136
Yes	3 (17.6)	14 (82.4)	
**Stage**			
Mild	NA	26	
Moderate	NA	21	
Severe	NA	14	


*^∗^*P*-values were calculated by *χ^*2*^* test*.

### Extraction and Characterization of Exosomes From Human Saliva

**FIGURE 3:**
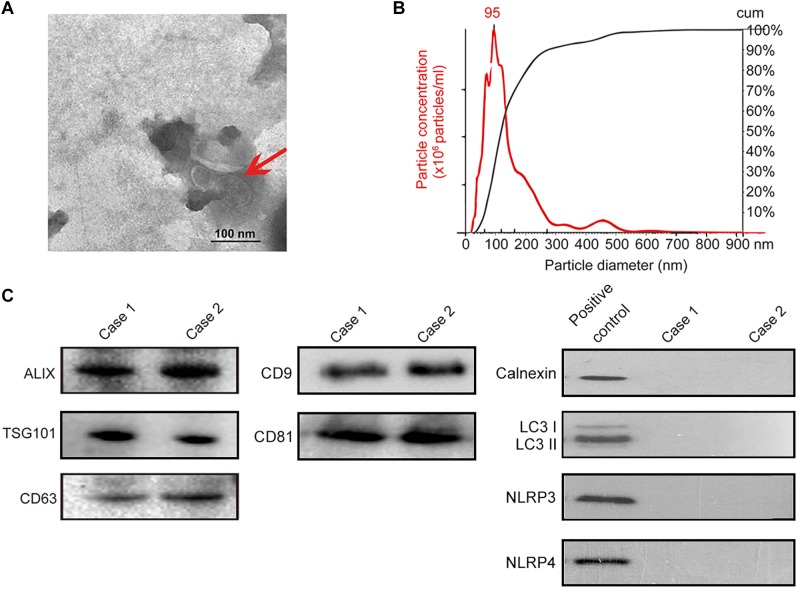
Identification of salivary exosomes in periodontitis patients and normal subjects. **(A)** Transmission electron microscopy of exosomes isolated from human saliva. Scale bar: 100 nm. **(B)** Exosomes concentration and size distribution by NanoSight analysis of human saliva. **(C)** Immunoblotting showed the exosomal membrane markers (ALIX, TSG101 CD63, CD9 and CD81), the intracellular protein Calnexin, the marker of autophagosome LC3 and markers of inflammasome (NLRP3 and NLRP4) in exosomes isolated from the saliva of one normal subject (case 01) and one periodontitis patient (case 02). Positive control for Calnexin was TE1 cells, and positive control for LC3 was TE1 cells after starvation for 6 h. Positive control for NLRP3 and NLRP4 was THP-1 cells.

Isolation of exosomes from human saliva was confirmed by transmission electron microscopy (TEM) (i.e., spherical membrane-bound particles with diameters between 30 and 100 nm) (Thery et al., 2009; Raposo and Stoorvogel, 2013; Figure 3A) and nanoparticle tracking analysis showing that human exosomes had an average diameter of 95 nm (Filipe et al., 2010; Webber and Clayton, 2013; Figure 3B). Immunoblotting of exosomal markers (ALIX, TSG101, CD63, CD9, CD81) and the intracellular protein that is not present in exosomes (Calnexin) (Mathivanan and Simpson, 2009; Taylor and Gercel-Taylor, 2011; Lotvall et al., 2014; Figure 3C) were performed for further confirmation. For eliminating the possibility of contamination by autophagosomes where significant amount of PD-L1 would be found, LC3 II, the marker of autophagosome was evaluated by immunoblotting; salivary exosomes samples did not contain LC3 while lysate of TE1 cells cultured in DMEM without serum for 6 h (positive control) showed the presence of LC3 II (Figure 3C). In addition, the marker of endoplasmic reticulum, calnexin, and the markers of inflammasomes were also analyzed by immunoblotting to exclude contamination of exosomes. The immunoblotting showed that calnexin, NLRP3 and NLRP4 could be detected in positive control but not in salivary exosomes samples (Figure 3C). Thus, exosomes were successfully isolated from saliva of periodontitis patients without significant contamination by autophagosomes, endoplasmic reticulum or inflammasomes.

### Salivary Exosomal *PD-L1* mRNA Was Elevated in Periodontitis Patients

**FIGURE 4:**
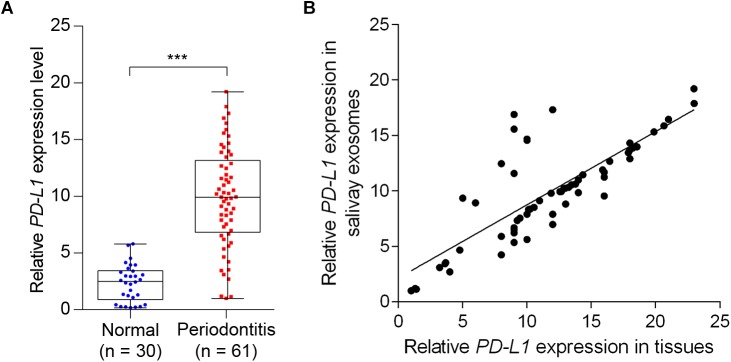
Detection of salivary exosomal *PD-L1* in periodontitis patients and normal subjects. **(A)** Salivary exosomal *PD-L1* was measured by RT-qPCR in periodontitis patients (*n* = 61) and normal controls (*n* = 30). Error bars indicate SEM. ^∗∗∗^*P <*0.001 by Student’s *t-*test. **(B)** Salivary exosomal *PD-L1* correlated with periodontal tissue *PD-L1* expression in periodontitis patients (Pearson’s correlation test).

We next determined the salivary exosomal and gingival *PD-L1* mRNA expression, in 61 periodontitis patients and 30 control subjects, by qPCR analysis. As shown in Figure 4A, 45 of 61 cases (73.8%) had increased salivary exosomal *PD-L1* expression compared with the mean salivary exosomal *PD-L1* expression in control subjects. Mean salivary exosomal *PD-L1* expression in the periodontitis patients was found to be about 10-fold higher when compared with the paired control subjects (*P*< 0.001). Similarly, mean gingival *PD-L1* mRNA expression in the periodontitis patients was more than 10-fold higher than that in control subjects (Figure 4B, *P*< 0.001). More importantly, salivary exosomal *PD-L1* mRNA levels highly correlated with gingival *PD-L1* mRNA levels in periodontitis patients (*r* = 0.800 and *P*< 0.001, Pearson’s correlation test; Figure 4B). Collectively, our results strongly suggested that salivary exosomal *PD-L1* mRNA could be a feasible biomarker of periodontitis.

### Clinical Relevance of Salivary Exosomal *PD-L1* mRNA in Periodontitis Patients

We further assessed the association between salivary exosomal *PD-L1* mRNA and clinical parameters in 61 periodontitis patients. The median value (i.e., relative value of salivary exosomal *PD-L1* mRNA = 9.90) was chosen to classify patients into high-(*n* = 31) and low-*PD-L1* (*n* = 30) groups (Table 2). High *PD-L1* expression was only associated with advanced stage (*P* = 0.005; *χ^2^* test; Table 2), and *PD-L1* expression was not found to be statistically significantly associated with other clinicopathological parameters (Table 2). Our results demonstrated that salivary exosomal *PD-L1* mRNA could reflect the stage of periodontitis, suggesting that PD-L1 may be relevant to the disease progression.

**Table 2:** The clinicopathological characteristics related to *PD-L1* expression in periodontitis patients.

Variables	No. of patients	*PD-L1* expression		*P–*value^∗^
		Low, *n* (%)	High, *n* (%)	
Total samples	61	30 (49.2)	31 (50.8)	
**Age (years)**				
≥50	39	18 (46.2)	21 (53.8)	0.529
<50	22	12 (54.5)	10 (45.5)	
**Gender**				
Female	32	19 (59.4)	13 (40.6)	0.653
Male	29	11 (37.9)	18 (62.1)	
**Tobacco use**				
No	40	21 (52.5)	19 (47.5)	0.474
Yes	21	9 (42.9)	12 (57.1)	
**Alcohol use**				
No	45	25 (55.6)	20 (44.4)	0.095
Yes	16	5 (31.3)	11 (68.8)	
**Hypertension**				
No	41	22 (53.7)	19(46.3)	0.316
Yes	20	8 (40.0)	12 (60.0)	
**Diabetes**				
No	47	25 (53.2)	22 (46.8)	0.251
Yes	14	5 (35.7)	9 (64.3)	
**Stage**				
Mild	26	19 (73.1)	7 (26.9)	0.005
Moderate	21	7 (33.3)	14 (66.7)	
Severe	14	4 (28.6)	10 (71.4)	


*^∗^*P*–values were calculated by *χ^*2*^* test. High in this analysis is based on a *PD-L1* level > 9.9 (median); the remaining individuals were classified as low*.

## Discussion

We characterized *PD-L1* mRNA expression in exosomes derived from saliva of periodontitis patients, and have evaluated the clinical relevance of the levels of salivary exosomes *PD-L1* mRNA in the disease. One of the main findings was that the level of salivary exosomes *PD-L1* mRNA in periodontitis patients is highly distinct relative to non-periodontitis controls. Moreover, high level of salivary exosomes *PD-L1* was associated with an advanced stage of periodontitis, suggesting it can reflect disease progression. This is the first to establish a procedure of detection of saliva-based exosomal *PD-L1* in disease, and the first salivary exosomal biomarker for periodontitis. In the majority of previous investigations of periodontitis biomarker, the specimens are either gingival tissues or gingival crevicular fluids (GCF) (Buduneli and Kinane, 2011; Ebersole et al., 2014; Jagannathan et al., 2014; Kebschull et al., 2014). However, sample collection and procedures for both gingival tissues and GCF are challenging: gingival tissue biopsy involves acquisition from invasive and limited tissue, and GCF sample collection involves sampling of a minute amount of fluid on filter paper strips, which requires a longer sampling time. Saliva-based assay overcomes these forgoing problems, as shown by our current study which involves an easy, non-invasive, and rapid collection of salivary specimens. We also demonstrate that mRNA can be extracted well from salivary exosomes, supporting the notion that exosomes-derived samples prevent mRNA from degradation (Kim et al., 2005; Soung et al., 2017). To our knowledge, this is the first to detect *PD-L1* in exosomes and in saliva in periodontitis. The procedure described in the current study may be used for detecting mRNAs in oral diseases as well as in other systemic diseases.

PD-L1 has been reported to play an important role in a wide range of cancers and inflammation-originated diseases including periodontitis (Ota et al., 2015; Clark et al., 2016; Concha-Benavente et al., 2016; Zhang et al., 2016; Groeger et al., 2017). The role of PD-L1 to inhibit destruction of inflammatory tissues has been reported (Scandiuzzi et al., 2014). In two previous reports, it has been shown that PD-L1 expression in periodontitis tissues is increased in mild and moderate periodontitis (Yuan et al., 2015; Zhang et al., 2016). However, it is unclear whether PD-L1 expression correlates with disease status of chronic periodontitis. It has been also reported that expression of PD-1 and PD-L1 in peripheral CD4^+^ T lymphocytes and CD8^+^ T lymphocyte of chronic periodontitis patients was upregulated (Zhu et al., 2014). High expression of PD-L1 in host cells may contribute to the chronicity of inflammatory disorders (Zamani et al., 2016). The previous work showed that the PD-L1 expression in different compartments may be different and have different roles in inflammatory disease. Therefore, further correlation between PD-L1 and periodontitis are worthy to investigated. In our current study, it suggests that assay of exosome-based *PD-L1* mRNA in saliva has potential to distinguish periodontitis from the healthy, and the levels correlate with the severity/stage of periodontitis. The increased expression of PD-L1 in severe periodontitis might due to the requirement of the body for more PD-L1 to inhibit destruction of inflammatory tissues. It has been shown that tumor cells upregulate PD-L1 expression to evade host immune response and thereby to maintain disease (Flies et al., 2016; Wang et al., 2016; Lakin et al., 2017). Moreover, the potential for PD-L1 to serve as a disease biomarker has also been revealed (Colwell, 2015; Fusi et al., 2015). PD-L1 is upregulated in cancers, such as glioblastoma (Wang et al., 2016), ovarian cancer (Abiko et al., 2013), lung cancer and oral carcinoma (Kim et al., 2005; Ota et al., 2015), and upregulated PD-L1 predicts worse survival. In periodontitis, PD-L1 has been detected in gingival tissues, gingival crevicular fluid (GCF) and blood (Olsen et al., 2016; Zhang et al., 2016; Groeger et al., 2017). Our analysis of published database validated that periodontitis specimens harbor high levels of PD-L1. Although detection of PD-L1 has been widely reported in tissues and blood (Fusi et al., 2015; Wang et al., 2016; Groeger et al., 2017), there are no publications for PD-L1 detection in salivary exosomes. We show that PD-L1 is present in exosomes of saliva from periodontitis patients, suggesting the possibility that PD-L1 is enriched in exosomes. Our results are consistent with a recent study that exosomes released from melanoma cells were found to carry a remarkable amount of PD-L1 on their surfaces (Chen et al., 2018). We here described a more convenient procedure for isolation and detection of PD-L1 in salivary exosomes. Since PD-L1 involves many diseases including cancers (i.e., oral cancers) and immune diseases (Gianchecchi et al., 2013; Dai et al., 2014; Roemer et al., 2016), the protocol established in current study may provide a platform for easy and rapid assay of PD-L1 in variety of applications. The limitation of the study was the relatively small size of the patient cohorts. Further investigations using larger and multiple cohorts are worthy to be performed to validate the findings of the current studies.

In summary, we described the procedure for isolating mRNA from exosomes derived from saliva of periodontitis patients. Our further assay showed that exosomal *PD-L1* in saliva was enriched in periodontitis and was associated with advanced stage of disease. These findings are worthy to validate in further investigations with expanding samples.

## Author Contributions

HZ conceived and designed the experiments. JY and DY provided patients and the study materials. YL, KL, XX, ZY, HD, and ZJ performed the *in vitro* experiments about patient specimen analysis, bioinformatics assay, and analyzed data. JY, YL, XX, S-CY, and HZ interpreted data and wrote the manuscript. JY, S-CY, DY, and HZ contributed to discussion and reviewed the manuscript.

## Conflict of Interest Statement

The authors declare that the research was conducted in the absence of any commercial or financial relationships that could be construed as a potential conflict of interest.

## Footnotes

1 http://www.broadinstitute.org/gsea/
